# Second Harmonic Generation in Janus Transition Metal Chalcogenide Oxide Monolayers: A First-Principles Investigation

**DOI:** 10.3390/nano13142150

**Published:** 2023-07-24

**Authors:** Peng Su, Han Ye, Naizhang Sun, Shining Liu, Hu Zhang

**Affiliations:** State Key Laboratory of Information Photonics and Optical Communications, Beijing University of Posts and Telecommunications, Beijing 100876, China; pengsu519@bupt.edu.cn (P.S.); snz@bupt.edu.cn (N.S.); liushining@bupt.edu.cn (S.L.)

**Keywords:** transition metal chalcogenide oxide, Janus structure, optical response, second harmonic generation

## Abstract

Due to the unique optical responses induced by vertical atomic asymmetry inside a monolayer, two-dimensional Janus structures have been conceived as promising building blocks for nanoscale optical devices. In this paper, second harmonic generation (SHG) in Janus transition metal chalcogenide oxide monolayers is systematically investigated by the first-principles calculations. Second-order nonlinear susceptibilities are theoretically determined for Janus MXO (M = Mo/W, X = S/Se/Te) monolayers. The calculated values are comparable in magnitude with Janus MoSSe monolayer. X-M-O symmetry breaking leads to non-zero components in vertical direction, compared with the non-Janus structure. Focusing on the SHG induced by incident light at 1064 nm, polarization-dependent responses of six Janus MXO monolayers are demonstrated. The symmetry of p-polarization changes from six-fold to three-fold with acute incidence angle. Moreover, the effects of biaxial strain on band structures and SHG are further investigated, taking MoSO as an exemplary case. We expect these results to bring in recipes for designing nonlinear optical devices based on Janus transition metal chalcogenide oxide monolayers.

## 1. Introduction

Nonlinear optics, an essential discipline for regulating and controlling light, have made incredible advances in theory and application [1,2,3]. Nonlinear optics are widely used in laser frequency conversion, optical communications, and optical computing [4,5]. However, nonlinear optical devices also face the challenge of integration and miniaturization. With the continuous development of nanotechnology, two-dimensional (2D) materials have shown extraordinary properties in nonlinear optics [6,7]. Mikhailov et al. investigated the nonlinear high-harmonic conversion efficiency and optical properties of graphene from the perspective of the electromagnetics and linear response theory. They discovered that graphene exhibited a significantly higher efficiency in high-harmonic conversion at terahertz frequencies, due to its non-harmonic, charge–carrier dispersion relationship [8]. Cox et al. investigated the nonlinear polarization response of graphene nanosheets near the plasmonic response wavelength of graphene using a combination of a tight-binding model and motion equations of a density matrix. They employed a classical model of nonharmonic oscillators to describe the polarization response under the influence of external optical field intensity. The study revealed that graphene nanodots exhibited several orders of magnitude that were higher nonlinear second-order and third-order polarization responses compared to metallic nanoparticles of similar dimensions [7].

Among the many 2D materials, transition metal dichalcogenides (TMDs) have received much attention. Malard et al. conducted measurements of the second harmonic generation (SHG) spectrum of MoS_2_ using femtosecond pulses. Their experimental results revealed that the SHG susceptibility of MoS_2_ monolayer reached a maximum value of 13 × 10^4^ pm^2^/V when the photonic energy ranged from 2 eV to 4 eV [9]. In the same year, Li et al. experimentally measured the optical response of MoS_2_ monolayer and a strong SHG was observed [10]. Since then, other studies have come to the same conclusion [11]. Moreover, MoS_2_ showed an intense nonlinear optical behavior in the third harmonic [12] and four-wave mixing [13,14]. Furthermore, Murray et al. experimentally investigated SHG in WS_2_ with synthetically or focused ion beam-induced structural defects. The results demonstrated the significant SHG capability of WS_2_ monolayer and that, despite the potential defects having a relatively minor impact on the second-order nonlinearity, photoluminescence (PL) is significantly affected [15].

However, due to the D_3h_ symmetry of MoS_2_ (WS_2_) monolayer, they do not possess $\chi_{\;}^{\left(2\right)}$ related to the vertical direction. In recent years, the optical properties of two-dimensional materials with broken symmetry structures have gradually attracted attention. Zhang et al. successfully synthesized the Janus MoSSe monomolecular layer based on MoSe_2_ by employing a controlled sulfide method to make S atoms substitute Se atoms in one layer, while Se atoms in the other layer remained unaltered [16]. The newly synthesized (2D) material breaks the out-of-plane symmetry of TMDs, converting the initial higher symmetry point group of D_3h_ to the lower symmetry point group, C_3V_, resulting in a host of excellent physical properties. For instance, the built-in dipole moment enhances the wave function separation of electron and hole, reinforcing the electron−phonon interactions, and ultimately leads to the longer exciton radiative recombination lifetime [17]. The Janus MoSSe nanoribbon with spontaneous curling can further reduce symmetry [18,19,20]. Sun et al. conducted quantum transport simulations to investigate the generation of photogalvanic effect (PGE) photocurrent in six Janus transition metal dichalcogenide (TMD) compounds. Under the influence of spontaneous curvature, the device symmetry is reduced from C_3v_ symmetry to C_s_ symmetry. The PGE increases in the zigzag and armchair directions for Janus TMD photodetectors with non-collinear electrodes [21]. Furthermore, it has been shown that the optical signal can be modified by introducing a different atom into an extended/molecular system [22,23,24]. Mocci et al. reported that the absorption spectra of C_24_H_12_ and C_32_H_14_ can be modified by substituting Si atoms in them [22]. The second-order nonlinear susceptibility strongly depends on the symmetry of crystals [25]. This means that the inherent low symmetry of Janus TMDs provides a platform for strong SHG. Two-dimensional materials avoid the requirement for phase matching because their crystal thickness is far less than the coherence length [26]. The second harmonic effect of Janus MoSSe had been investigated by first-principles calculations, which discovered the extraordinary nonlinear optical responses of Janus MoSSe with monolayer and bilayer heterostructures [27,28]. Meanwhile, Bian et al. conducted experimental measurements to investigate the power and wavelength dependence of SHG in MoSSe monolayer. By tuning the excitation wavelength, they discovered that the enhancement of SHG coincided with the energy of the C-exciton state in the linear absorption spectrum of monolayer MoSSe. Therefore, it is inferred that the interaction between light and matter is particularly strong when there is resonance between two-photon transitions and the C-exciton state, resulting in a large second-order susceptibility. The Janus MoSSe monolayer is a tunable nonlinear medium in two-dimensional limits, with the potential for on-chip, frequency-doubling applications [29]. And Li et al. investigated the nonlinear optical properties of MoSSe nanosheets using the open-aperture Z-scan technique. The results showed that the nonlinear optical response of MoSSe in the visible range was superior to that in the near-infrared range [30].

Like the Janus TMD system, the phonon spectra of the MXO (M = W/Mo, X = S/Te/Se) monolayer indicates that their structures are stable [31,32], and the MXO monolayers also show excellent optoelectronic properties [31,32,33,34,35]. Varjovi et al. investigated three structural phases (1H, 1T, and 1T′) of Janus WXO (X = S, Se, and Te) monolayers and studied their vibrational, thermal, elastic, piezoelectric, and electronic properties using first-principles methods. The results demonstrated the multifunctional mechanical and electronic properties of Janus WXO monolayers, along with a large piezoelectric response [32]. Recently, Xu et al. oxidized monolayer MoS_2_ by lowering the concentration of H_2_O_2_, which can form the monolayer of Janus MoSO [36]. In addition, Kang et al. and Shioya et al. also proposed the oxidation of single-molecule films WX_2_ (X = S/Se) using ultraviolet-ozone and laser heating methods, respectively [37,38]. Nguyen et al. conducted a comprehensive study on the tunable electronic and magnetic properties of monolayer MoSO based on density functional theory (DFT) [34]. Additionally, Li et al. identified Janus MoSO monolayer as a potential candidate material for optoelectronic applications [31]. Waheed et al. found that MoSO had an absorption efficiency of up to 90% in the infrared to the ultraviolet region of the spectrum, so MoSO can be an effective candidate for photocatalysis and solar cells [39]. Falahati et al. investigated the optical properties of Janus WSO monolayer using first-principle calculations based on DFT. The findings suggested that the Janus WSO monolayer presents new opportunities for electronic, optoelectronic, and photonic applications [40]. The physical properties of Janus MXOs have been studied in various aspects, but there is a gap in the research of Janus MXOs in the field of nonlinear optics.

In this paper, the second harmonic generation in Janus MXO (M = Mo/W, X = S/Se/Te) monolayers is investigated, based on first-principles calculations. The second-order nonlinear susceptibilities of MXO are obtained using Random-Phase Approximation (RPA) [41]. Our findings suggest that monolayer MXO provides a potential platform for nonlinear optics. Furthermore, we study the biaxial strain effect on the bandgap and SHG of Janus MoSO monolayer. The computational results indicate that second-order nonlinear susceptibility of Janus MoSO monolayer exhibits strain dependence under biaxial strain.

## 2. Methods

All calculations are performed using the PWmat package, which is a plane wave pseudopotential software based on density-functional theory with GPU acceleration [42,43]. The exchange-correlation function adopts generalized gradient approximation (GGA) of Perdew–Burke–Ernzerhof (PBE) [44]. The spin orbit coupling (SOC) is included in all property calculations. The plane wave cutoff energy of the wave function is set to 70 Ry. The vacuum layer of 30 Å is set in models to avoid the influence of periodic boundary conditions on the calculation. In the primitive cell optimization for Janus MXO, the conjugate gradient (CG) algorithm is used for atomic relaxation [45]. The convergence standard for structural relaxation is set 0.001 eV/Å for atomic forces and less than 0.02 eV/atom for lattice stresses, and van der Waals interactions are considered by the DFT-D3 method [46]. For the structure optimizations and band structure calculations, we set the Monkhost-Pack k-point sampling grid to 15 × 15 × 1. In addition, to avoid the impact of the bandgap underestimation caused by the PBE form, we also adopt the Heyd–Scuseria–Ernzerhof (HSE06) heterogeneous generalization function in the band structure calculations [47].

For calculating nonlinear optical properties, we use the RPA-based method SHG calculation package developed in-house by PWmat [48]. Calculating the nonlinear optical properties requires a dense k-point grid. After testing, we use Monkhost-Pack k-point sampling with a 70 × 70 × 1 grid and 140 electron energy band numbers. We sample 20,000 energy points in the range of 0–6 eV. Methods dealing with bandgap underestimation in the calculation of optical properties include GW, Bethe-Salpeter equation (BSE) and scissor operators, etc. [28,49,50,51]. We adopt the scissor operator to overcome the bandgap underestimation problem caused by the PBE, and the HSE calculation results provide a reference for the correction value of the scissor operator. The expression of second-order nonlinear susceptibility ($\chi^{abc}\left(-2\omega ,\omega ,\omega \right)$) was derived by Sharma [52]. Since the calculation method is based on RPA, exciton effects are not included [53]. The second-order susceptibility is contributed by interband transitions, intraband transitions and modulation of interband terms by intraband terms, which can be described as [48,52]

(1) $$\chi_{\mathrm{inter}}^{abc}(2\omega ,\omega ,\omega )=\frac{e^{3}}{\hbar^{2}}\sum_{nml}^{\prime }\int \frac{dk}{4\pi^{3}}\frac{r_{nm}^{a}\left\{r_{ml}^{b}r_{ln}^{c}\right\}}{\left(\omega_{ln}-\omega_{ml}\right)}\left\{\frac{2f_{nm}}{\left(\omega_{mn}-2\omega \right)}+\frac{f_{ml}}{\left(\omega_{ml}-\omega \right)}+\frac{f_{ln}}{\left(\omega_{ln}-\omega \right)}\right\}$$

(2) $$\begin{array}{cc} \chi_{\mathrm{intra}\;}^{abc}(2\omega ,\omega ,\omega )= & \frac{e^{3}}{\hbar^{2}}\int \frac{dk}{4\pi^{3}}[\sum_{nml}^{\prime }\omega_{mn}r_{nm}^{a}\{r_{ml}^{b}r_{ln}^{c}\}\{\frac{f_{nl}}{\omega_{ln}^{2}(\omega_{ln}-\omega )}-\frac{f_{lm}}{\omega_{ml}^{2}(\omega_{ml}-\omega )}\}-8i\sum_{nm}^{\prime }\frac{f_{nm}r_{nm}^{a}\{\Delta_{mn}^{b}r_{mn}^{c}\}}{\omega_{mn}^{2}(\omega_{mn}-2\omega )}\\ & +2\sum_{nml}^{\prime }\frac{f_{nm}r_{nm}^{a}\{r_{ml}^{b}r_{ln}^{c}\}(\omega_{ml}-\omega_{ln})}{\omega_{mn}^{2}(\omega_{mn}-2\omega )}] \end{array}$$

(3) $$\chi_{\mathrm{mod}}^{abc}(2\omega ,\omega ,\omega )=\frac{e^{3}}{2\hbar^{2}}\int \frac{dk}{4\pi^{3}}[\sum_{nml}^{\prime }\frac{f_{nm}}{\omega_{mn}^{2}\left(\omega_{mn}-\omega \right)}\left\{\omega_{nl}r_{lm}^{a}\left\{r_{mn}^{b}r_{nl}^{c}\right\}-\omega_{lm}r_{nl}^{a}\left\{r_{lm}^{b}r_{mn}^{c}\right\}\right\}+i\sum_{nm}^{\prime }\frac{f_{nm}r_{nm}^{a}\left\{r_{mn}^{b}\Delta_{mn}^{c}\right\}}{\omega_{mn}^{2}\left(\omega_{mn}-\omega \right)}]$$

where

$$r_{nm}^{a}(k)=\frac{p_{nm}^{a}(k)}{im\omega_{nm}(k)}$$

$$\Delta_{nm}^{a}(k)=v_{nn}^{a}(k)-v_{mm}^{a}(k)$$

In order to avoid the effect of vacuum layer thickness, we multiply the results of the in-plane component ($\left|\chi_{yyy}^{\left(2\right)}\right|$,$\left|\chi_{xxz}^{\left(2\right)}\right|$) by a factor φ, which is the ratio of the vacuum layer thickness *c* to the atomic thickness *t*. For the out-of-plane components ($\left|\chi_{zxx}^{\left(2\right)}\right|$,$\left|\chi_{zzz}^{\left(2\right)}\right|$), we obtain $\left|\chi_{abs}^{\left(2\right)}\right|$ by multiplying $\chi_{real}^{\left(2\right)}$ by 1/φ and $\chi_{imag}^{\left(2\right)}$ by φ [54,55,56,57].

## 3. Results and Discussion

**Figure 1:**
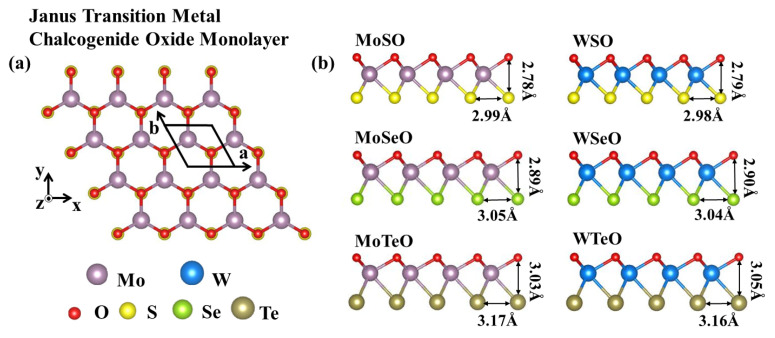
(**a**) Janus MXO monolayers with (**a**) top view and (**b**) side view.

**Figure 2:**
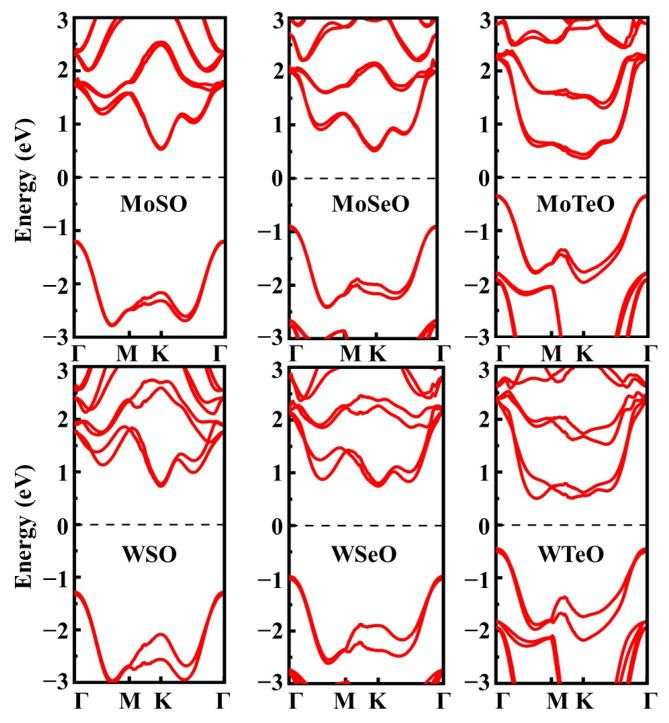
Band structures of Janus MXO monolayers calculated with HSE06 + SOC.

As shown in Figure 1, the Janus MXO (M = Mo/W, X = S/Se/Te) monolayer is a hexagonal structure with C_3v_ symmetry. The transition metal atom layer is sandwiched between the oxygen atom layer and the sulfur group atom layer. The theoretically optimized structural parameters are summarized in Table 1. The results show that the thickness of crystals increases with the increase of the X-atom radius, and similarly, the size of the atomic radius affects the lattice constant. The lengths of W-O and Mo-O bond remain almost the same in all systems. Our results are also compared with literatures to verify the reliability of our method. The van der Waals interaction is considered in the calculations. As an example, the optimized lattice parameter of MoSeO is 3.05 Å. Since the radius of the O atom is smaller than that of the Se atom, the lattice constant is smaller than that of MoSe_2_ [58]. Moreover, we investigate the electronic properties of the Janus MXO family. The Fermi level is set to zero. The band structures from PBE are shown in Figure S1. To reduce the underestimation of PBE bandgap, we resort to HSE06. As shown in Figure 2, the results indicate that all six monolayers are indirect-gap semiconductors, and their bandgaps are between MX_2_ and MO_2_. The bandgap of Janus WSO is 1.62 eV, which locates between WO_2_ (1.52 eV) [58] and WS_2_ (1.81 eV) [59]. The valance band maximums (VBM) are all located at the Γ point. The conduction band minimum (CBM) of MoTeO is located between Γ and M points, while the CBM of the other five materials is located on the K point. When spin-orbit coupling (SOC) is considered, the valence band edge of MoSO monolayer shows an energy split of 153 meV at the K-high symmetry point, 172 meV for MoSeO monolayer, 193 meV for MoTeO monolayer, 474 meV for WSO monolayer, 473 meV for WSeO monolayer and 447 meV for WTeO monolayer. However, they all split little at the Γ point. In SHG calculation, we use the difference between the bandgap of PBE and HSE06 as a scissor correction. Thus, the scissor operators for the MXO monolayers are 0.56 eV (MoSO), 0.27 eV (MoSeO), 0.47 eV (MoTeO), 0.39 eV (WSO), 0.37 eV (WSeO) and 0.41 eV (WTeO), respectively.

**Figure 3:**
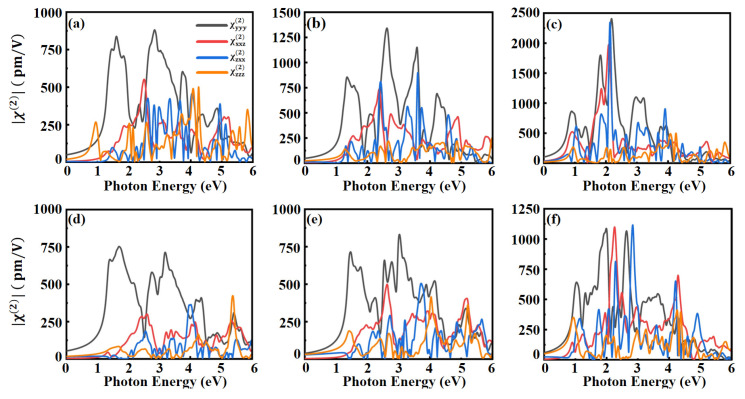
Second-order nonlinear susceptibility $\chi_{yyy}^{\left(2\right)}$ (black line), $\chi_{xxz}^{\left(2\right)}$ (red line), $\chi_{zxx}^{\left(2\right)}$ (blue line), $\chi_{zzz}^{\left(2\right)}$ (orange line) for (**a**) MoSO, (**b**) MoSeO, (**c**) MoTeO, (**d**) WSO, (**e**) WSeO and (**f**) WTeO.

Considering that the crystal symmetry of the Janus MXO monolayers is C_3V_, there are eleven nonzero components of the second-order nonlinear susceptibility: $\chi_{yxx}^{\left(2\right)}=\chi_{xxy}^{\left(2\right)}=\chi_{xyx}^{\left(2\right)}={-\chi }_{yyy}^{\left(2\right)}$, $\chi_{xxz}^{\left(2\right)}=\chi_{xzx}^{\left(2\right)}=\chi_{yyz}^{\left(2\right)}=\chi_{yzy}^{\left(2\right)}$, $\chi_{zxx}^{\left(2\right)}=\chi_{zyy}^{\left(2\right)}$ and $\chi_{zzz}^{\left(2\right)}$, as shown in Figure 3. The largest $\left|\chi_{yyy}^{\left(2\right)}\right|$ for the Janus MXO monolayer is caused by materials with minimal bandgap energy and parallel electronic band. The similar trend is also observed in Janus TMDs monolayer [60]. The results show that the principal polarization component $\left|\chi_{yyy}^{\left(2\right)}\right|$ generally increases with the increase of atomic number of the sulfur level in the following order: MoSO (WSO) < MoSeO (WSeO) < MoTeO (WTeO). The second-order harmonic response of the Mo-based monolayer is greater than that of W-based monolayer, while the sulfur group elements are identical. The peak position of $\left|\chi_{yyy}^{\left(2\right)}\right|$ is red shifted with the increase of the atomic number of X and the maximum of $\left|\chi_{yyy}^{\left(2\right)}\right|$ increases. For the Mo-based monolayers, the first peaks of $\left|\chi_{yyy}^{\left(2\right)}\right|$ of MoSO, MoSeO, MoTeO locate at 1.46 eV, 1.35 eV and 0.9 eV, respectively. For the W-based monolayers, the first peaks of WSO, WSeO, and WTeO locate at 1.70 eV, 1.46 eV, and 1.02 eV, respectively. Moreover, the Janus structure breaks the out-of-plane symmetry, resulting in larger SHG in the out-of-plane direction, namely $\chi_{zxx}^{\left(2\right)}$,$\chi_{zyy}^{\left(2\right)}$, and $\chi_{zzz}^{\left(2\right)}$. As illustrated in Figure 3a–c, the peak values of $\left|\chi_{zxx}^{\left(2\right)}\right|$ of MoSO, MoSeO and MoTeO are 420 pm/V, 900 pm/V, 2346 pm/V, respectively. The susceptibility $\left|\chi_{zxx}^{\left(2\right)}\right|$ of MoTeO monolayer is almost five times higher than that of MoSO monolayer. In Figure 3d–f, the peak of susceptibility $\left|\chi_{zxx}^{\left(2\right)}\right|$ of W-base is relatively lower than that of Mo-base, with only 364 pm/V, 505 pm/V, 1116 pm/V for WSO, WSeO, and WTeO, respectively. It is worth noting that the charge transfer is not uniform due to the difference in electronegativity of the elements, which results in the largest intensity of the second-order susceptibility in all directions for MoTeO monolayer. The out-of-plane polarization component $\left|\chi_{zxx}^{\left(2\right)}\right|$ exceeds the in-plane component $\left|\chi_{yyy}^{\left(2\right)}\right|$ when the photon energy reaches about 2 eV, indicating that MoTeO monolayer gives rise to the potential applications for NLO devices. In addition, we compare the results of SHG of MoSSe monolayer calculations with other work [27,28,61,62], as shown in Figure S2. The first peak of the MoSSe monolayer is located near 0.9 eV. The MoSSe monolayer has a maximum magnitude of 1700 pm/V for $\left|\chi_{yyy}^{\left(2\right)}\right|$ and 550 pm/V for $\left|\chi_{zxx}^{\left(2\right)}\right|$, which is consistent with what has been reported in other work. In terms of the SHG magnitude of MoXO, MoSO < MoSeO < MoTeO. Based on this, we take MoSSe as a benchmark and further investigated the SHG in the MoSO monolayer and MoTeO monolayer as two representatives, and compared them with MoSSe. Considering a wavelength of 1064 nm laser, Janus MoSO monolayer ($\left|\chi_{yyy}^{\left(2\right)}\right|\approx 0.5\;\times$ MoSSe, $\left|\chi_{zxx}^{\left(2\right)}\right|\approx 0.3\;\times \;MoSSe$) and Janus MoTeO monolayer ($\left|\chi_{yyy}^{\left(2\right)}\right|\approx 0.8\;\times$ MoSSe, $\left|\chi_{zxx}^{\left(2\right)}\right|\approx 16\;\times \;MoSSe$) show excellent out-of-plane responses and in-plane responses in SHG. The Janus transition metal chalcogenide oxide monolayers can play as a promising material platform to investigate SHG.

**Figure 4:**
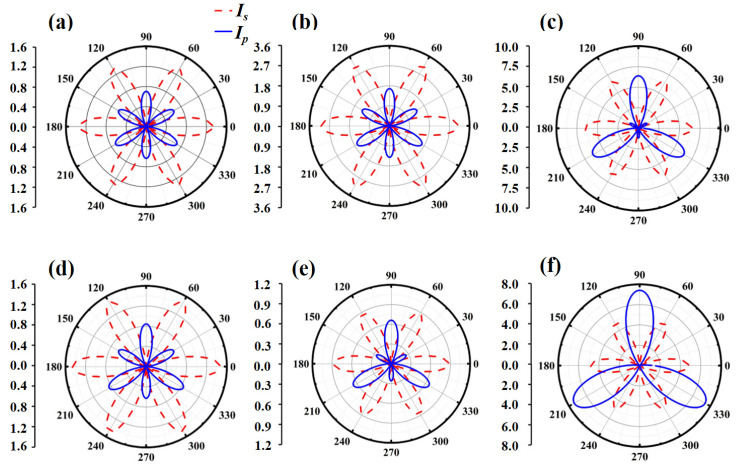
s- and p-polarization of nonlinear optical response of monolayer (**a**) MoSO, (**b**) MoSeO, (**c**) MoTeO, (**d**) WSO, (**e**) WSeO and (**f**) WTeO with respect to s-polarized incident light, the wavelengths of all cases are 1064 nm (1.16 eV). The incident angle is 45°.

According to the C_3v_ symmetry of Janus MXO, the two polarization components of nonlinear optical response can be expressed as [27]

(4) $$I_{s}\propto {\left(1/2\chi_{yyy}\cos3\phi \right)}^{2}I_{0}^{2}$$

(5) $$I_{p}\propto {\left(1/2\chi_{yyy}\cos\theta \sin3\phi -1/2\chi_{zyy}\sin\theta \right)}^{2}I_{0}^{2}$$

where θ is the angle between the incident light and the normal line of the monolayer plane, ϕ is the angle between the incident light and the zigzag direction of the in-plane Janus MXO. According to Equation (5), the polarization response is not only related to the polarization angle ϕ but also to the incident angle θ and magnitude of $\chi_{zyy}^{\left(2\right)}$. We set the angle of the incident light as 45° (θ=45°). When the wavelength of the incident laser is 1064 nm (1.16 eV), the magnitudes of $\chi_{yyy}^{\left(2\right)}$ and $\chi_{zyy}^{\left(2\right)}$ for Janus MoTeO monolayer are 5.11 × 10^2^ pm/V and 2.06 × 10^2^ pm/V, respectively. The generated polarizations for all Janus MXO monolayers are shown in Figure 4. The s-polarization has six-fold rotational symmetry while p-polarization possesses triple rotational symmetry. For the Janus MoTeO monolayer, it has the largest SHG intensity of polarization response among the studied Janus MXO. In addition, the response of s-polarization reaches a maximum and the p-polarization response is almost nonexistent for *ϕ* = 60°, which is determined by the magnitudes of $\chi_{yyy}^{\left(2\right)}$ and $\chi_{zyy}^{\left(2\right)}$. It should be noted the intensities of s-polarization and p-polarization response vary at different incident light wavelengths.

**Figure 5:**
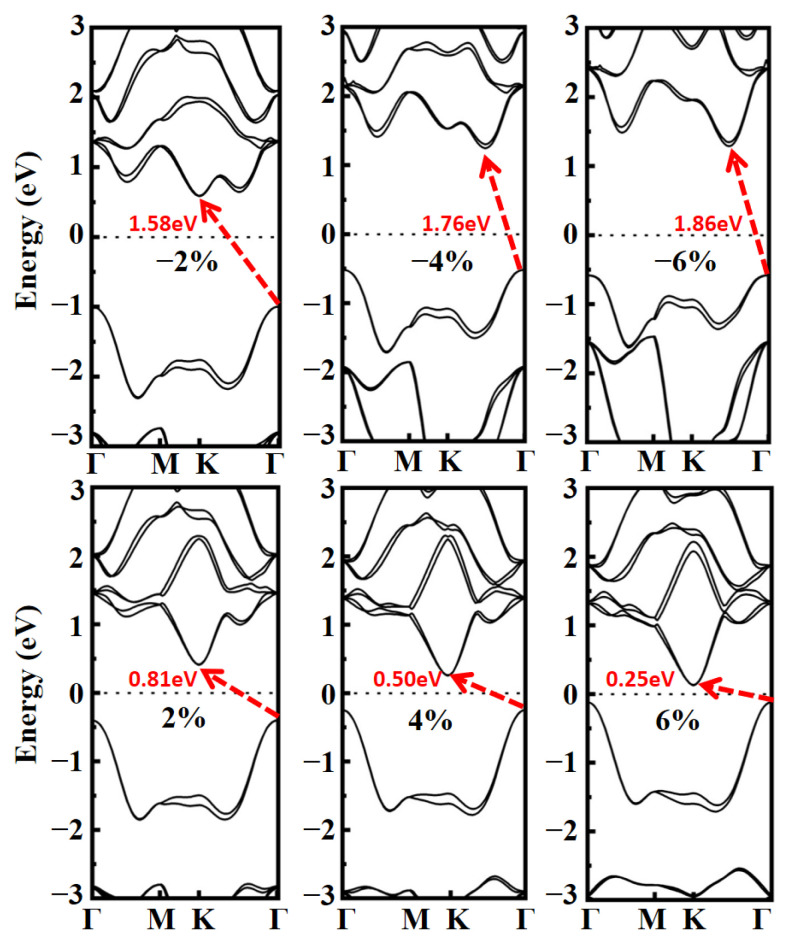
Band structures of MoSO monolayer under biaxial strains.

Next, the impact of biaxial tensile (compressive) strain is investigated, taking the Janus MoSO monolayer as an exemplary case. In our work, biaxial strain is achieved by adjusting the lattice constants *a* and *b*. For structures under different strains, we fix the length of the cell in the *c*-direction and the lattice constants, and then, relax the positions of all atoms. For example, in the absence of strain, the lattice constants of MoSO monolayer are *a*_0_ = *b*_0_ = 2.987, and the atomic distance between Se and S is 2.78 Å. When a tensile strain of 2% is applied, we modify the lattice constants to *a* = *b* = 3.047, and the atomic distance between Mo and S is reduced to 2.75 Å as a result. The bandgaps of Janus MoSO monolayer under tensile and compressive strain are illustrated in Figure S3. The bandgap decreases considerably when the strain shifts from compression to tension. As can be seen from Figure 5, the bandgap decreases fastest when the strain is −2% to 2%. In addition, we find the CBM locates at the K point when 2% compressive strain is applied to MoSO monolayer. With the compressive strain increasing, the CBM gradually shifts from the K point to a point in K-Γ path and the energy at K point on the conduction band continues to move to higher value. Nevertheless, the VBM remains on Γ point. On the other hand, with the tensile strain increasing, the CBM and VBM remain locate at the K and Γ point, respectively. Meanwhile, the CBM and VBM respectively shift downward and upward, which clearly decreases the bandgap.

**Figure 6:**
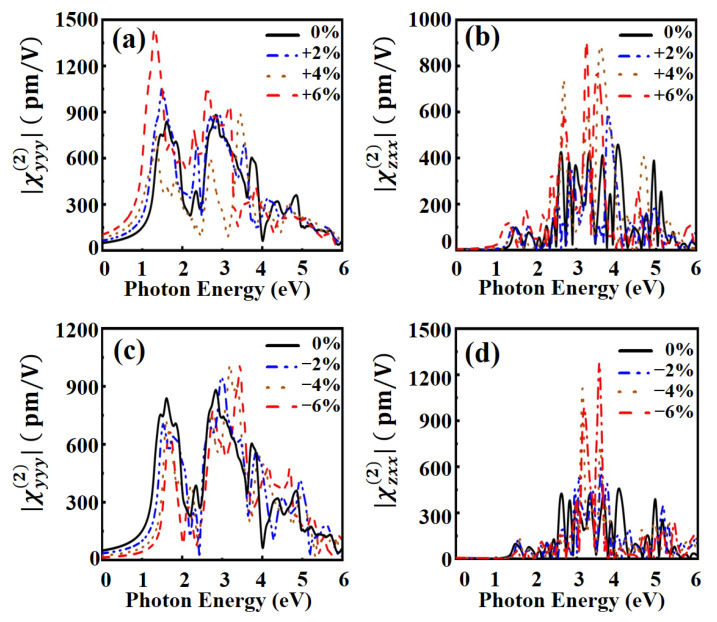
Second-order nonlinear susceptibility $\chi_{yyy}^{\left(2\right)}$ for MoSO under (**a**) tensile strain and (**c**) compressive strain. Second-order nonlinear susceptibility $\chi_{zxx}^{\left(2\right)}$ for MoSO under (**b**) tensile strain and (**d**) compressive strain.

We finally calculate the SHG under biaxial strain. In the absence strain, $\left|\chi_{yyy}^{\left(2\right)}\right|$ is the dominant component in the Janus MoSO monolayer, and the C_3V_ symmetry of Janus MoSO monolayer yields out-of-plane polarization components. Thus, we mainly calculate the $\left|\chi_{yyy}^{\left(2\right)}\right|$, $\;\left|\chi_{zxx}^{\left(2\right)}\right|$ and $\left|\chi_{zzz}^{\left(2\right)}\right|$ under strain. As shown in Figure 6, the bandgap of the band structure changes when strain is introduced, which results in a shift of all the peak positions of $\left|\chi_{yyy}^{\left(2\right)}\right|$. For example, in Figure 6a,c, the first peak position shifts 0.1 eV at 2% strain. As the strain increases, the peak shifts more. The magnitude of $\left|\chi_{yyy}^{\left(2\right)}\right|$ in the low-energy region increases remarkably with the increase of tensile strain. In Figure 6b,d, as the compressive strain increases, it causes an obvious increase in the magnitude of $\left|\chi_{zxx}^{\left(2\right)}\right|$ in photon energy range of 3–4 eV. However, the magnitude of $\left|\chi_{zxx}^{\left(2\right)}\right|$ remained essentially constant in the low-energy region with the increase of compressive strain. Interestingly, by applying a 6% compressive strain, we observe that the peak magnitude of the out of plane component $\left|\chi_{zxx}^{\left(2\right)}\right|$ at 3.5 eV is three times larger than that of the in-plane component $\left|\chi_{yyy}^{\left(2\right)}\right|$. The other two independent components ($\left|\chi_{xxz}^{\left(2\right)}\right|$, $\left|\chi_{zzz}^{\left(2\right)}\right|$) are shown in Figure S4. Applying different strains result in significant changes in second-order nonlinear susceptibility, which exhibits a strong strain dependence.

## 4. Conclusions

Based on DFT, we investigate the structural and electronic properties of Janus MXO (M = Mo/W, X = S/Se/Te) monolayers and further the second harmonic generation. The results show the out-of-plane symmetry breaking of Janus MXO monolayer leads to non-zero second-order nonlinear susceptibilities in vertical direction. Among these materials, a larger out-of-plane component is produced compare to the Janus MoSSe monolayer. In addition, we calculate the s- and p-polarization of nonlinear optical response of all Janus MXO monolayers. The symmetry of p-polarization changes from six-fold to three-fold with acute incidence angle. We also study the effect of biaxial strain on the band structures and SHG in Janus MoSO monolayer. The bandgap can be tuned by applying biaxial strain and we observe the four independent components of nonlinear susceptibility with different variations. By applying compressive strain, the out of plane component $\left|\chi_{zxx}^{\left(2\right)}\right|$ have peaks exceeding the in-plane component $\left|\chi_{yyy}^{\left(2\right)}\right|$ within the range of 3–4 eV photon energy. In this sense, the SHG in MoSO monolayer exhibits sensitivity to strain. The results show large SHG in Janus transition metal chalcogenide oxide monolayers and this material family can be a potential platform for nanoscale nonlinear optical devices.

## Figures and Tables

**Table 1:** Lattice parameters and bandgaps of Janus MXO monolayers.

This Work				Other Works			
	Lattice Parameter (Å)	Bandgap (eV)		Lattice Parameter (Å)	Bandgap (eV)		
	*a, b*	PBE	HSE	*a, b*	PBE	HSE	Ref.
MoSO	2.987	1.17	1.73	3.000	1.09	1.67	[39]
MoSeO	3.050	0.87	1.14	3.077	0.82	1.32	[33]
MoTeO	3.172	0.24	0.71	3.170	0.25	0.73	[31]
WSO	2.982	1.62	2.01	3.050	1.48	2.06	[60]
WSeO	3.042	1.34	1.71	3.071	1.32	1.89	[33]
WTeO	3.16	0.55	0.96	3.190	0.52	0.97	[60]

## Data Availability

The data presented in this study are available on request from the corresponding author.

## Supplementary Materials

The following supporting information can be downloaded at: https://www.mdpi.com/article/10.3390/nano13142150/s1, Figure S1. Band structures of Janus MXO monolayers calculated with PBE + SOC. Figure S2. Calculated second-order nonlinear susceptibility components of MoSSe (a) $\chi_{yyy}^{\left(2\right)}$, (b) $\chi_{xxz}^{\left(2\right)}$, (c) $\chi_{zxx}^{\left(2\right)}$, (d) $\chi_{zzz}^{\left(2\right)}$. Our calculated result (black line) are compared to Ref. [61] in green line, Ref. [28] in blue line, Ref. [27] in pink line, Ref. [60] in yellow line, Ref. [62] in purple line. Figure S3. Bandgap of Janus MoSO monolayer as a function of biaxial strain. Figure S4. Second-order nonlinear susceptibility ${\;\chi }_{zzz}^{\left(2\right)}$ for MoSO under (a) tensile strain and (c) compressive strain. Second-order nonlinear susceptibility $\chi_{xxz}^{\left(2\right)}$ for MoSO under (b) tensile strain and (d) compressive strain.
