# Supplementary material for: Second Harmonic Generation in Janus Transition Metal Chalcogenide Oxide Monolayers: A First-Principles Investigation
Source: Nanomaterials (Basel). 2023 Jul 24;13(14):2150. doi: 10.3390/nano13142150 (PMC10386494; doi:10.3390/nano13142150)
Supplement: Supplementary file 1 [file nanomaterials-13-02150-s001.zip › nanomaterials-2484502-supplementary.pdf]

Supporting Information

Second Harmonic Generation in Janus Transition Metal Chalcogenide Oxide Monolayers: A First-Principles Investigation

Peng Su, Han Ye *, Naizhang Sun, Shining Liu and Hu Zhang *

State Key Laboratory of Information Photonics and Optical Communications, Beijing University of Posts and Telecommunications, Beijing 100876, China; pengsu519@bupt.edu.cn (P.S.); snz@bupt.edu.cn (N.S.); liushining@bupt.edu.cn (S.L.)

* Correspondence: han_ye@bupt.edu.cn (H.Y.); zhh309@bupt.edu.cn (H.Z.)

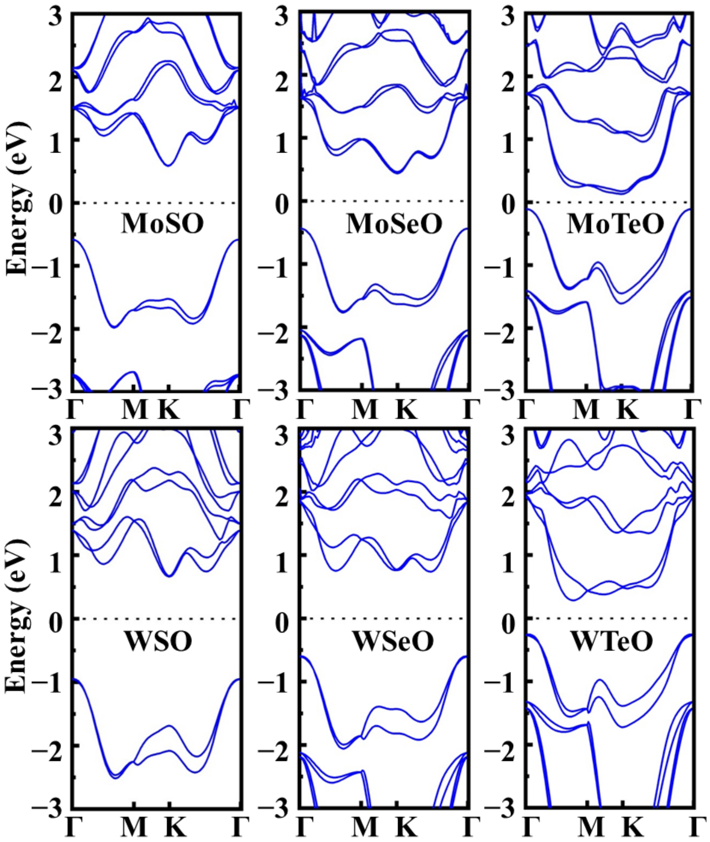

Figure S1. Band structures of Janus MXO monolayers calculated with PBE+SOC.

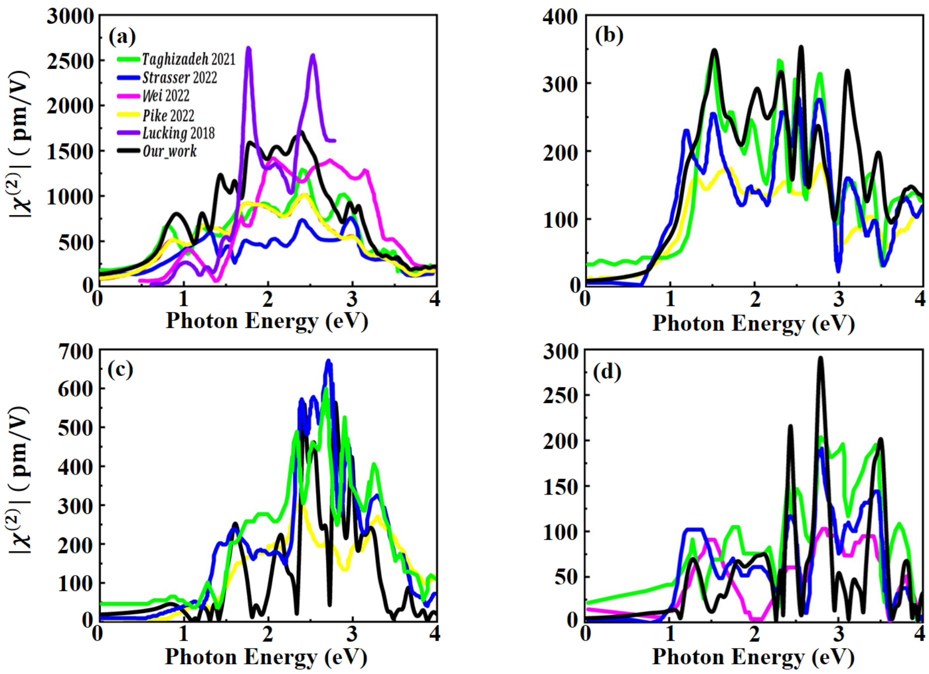

Figure S2. Calculated second-order nonlinear susceptibility components of MoSSe (a) $\chi^{(2)}_{yyy}$, (b) $\chi^{(2)}_{xxz}$, (c) $\chi^{(2)}_{zxx}$,(d) $\chi^{(2)}_{zzz}$. Our calculated result (black line) are compared to Ref. [61] in green line, Ref. [28] in blue line, Ref. [27] in pink line, Ref. [60] in yellow line, Ref. [62] in purple line.

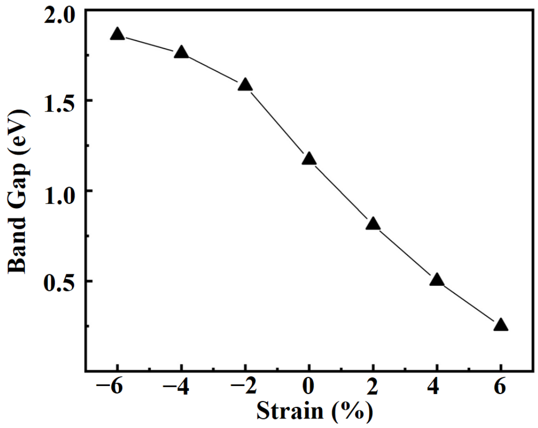

Figure S3. Bandgap of Janus MoSO monolayer as a function of biaxial strain.

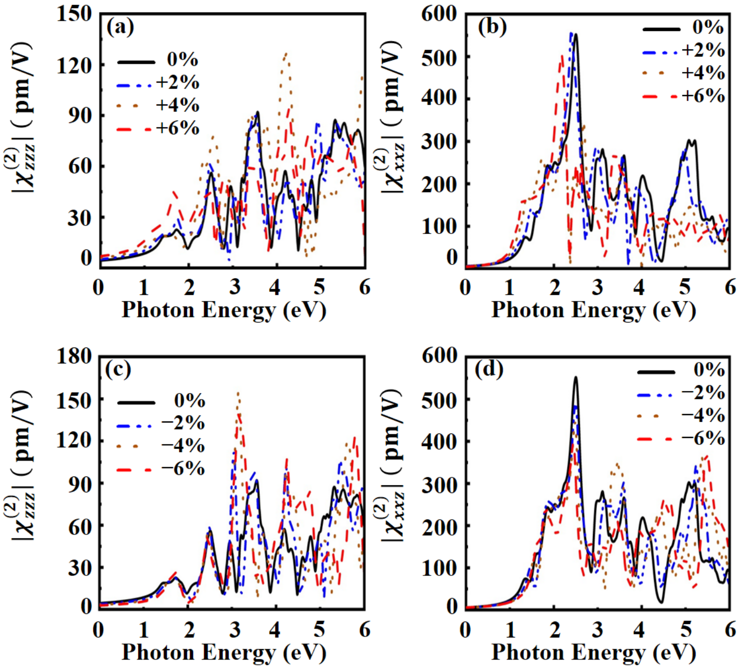

Figure S4. Second-order nonlinear susceptibility $\chi^{(2)}_{zzz}$ for MoSO under (a) tensile strain and (c) compressive strain. Second-order nonlinear susceptibility $\chi^{(2)}_{xxz}$ for MoSO under (b) tensile strain and (d) compressive strain.
